# Word recognition memory and serum levels of Borna disease virus specific circulating immune complexes in obsessive–compulsive disorder

**DOI:** 10.1186/s12888-022-04208-3

**Published:** 2022-09-08

**Authors:** Yuanyuan Zhang, A Alwin Prem Anand, Liv Bode, Hanns Ludwig, Hinderk M. Emrich, Detlef E. Dietrich

**Affiliations:** 1 Clinic of Psychiatry, Social Psychiatry and Psychotherapy, Hannover Medical School, 30625 Hanover, Germany; 2 Present Address: Social Psychiatry Counseling Center, Region Hannover, Podbielskistr. 157, 30177 Hanover, Germany; 3 Freelance Bornavirus Workgroup, Beerenstr. 41, 14163 Berlin, Germany; 4 AMEOS Klinikum Hildesheim, Goslarsche Landstr. 60, 31135 Hildesheim, Germany; 5 Center for Systems Neuroscience Hannover, Hanover, Germany

**Keywords:** Obsessive–compulsive disorder, Borna disease virus 1 (BoDV-1), Event-related brain potentials, Word recognition, Old/new effect, Impaired memory recollection process

## Abstract

**Background:**

Borna disease virus 1 (BoDV-1) is a non-segmented, negative-strand RNA virus that persistently infects mammals including humans. BoDV-1 worldwide occurring strains display highly conserved genomes with overlapping genetic signatures between those of either human or animal origin. BoDV-1 infection may cause behavioral and cognitive disturbances in animals but has also been found in human major depression and obsessive–compulsive disorder (OCD). However, the impact of BoDV-1 on memory functions in OCD is unknown.

**Method:**

To evaluate the cognitive impact of BoDV-1 in OCD, event-related brain potentials (ERPs) were recorded in a continuous word recognition paradigm in OCD patients (*n* = 16) and in healthy controls (*n* = 12). According to the presence of BoDV-1-specific circulating immune complexes (CIC), they were divided into two groups, namely group H (high) and L (low), *n* = 8 each. Typically, ERPs to repeated items are characterized by more positive waveforms beginning approximately 250 ms post-stimulus. This “old/new effect” has been shown to be relevant for memory processing. The early old/new effect (ca. 300–500 ms) with a frontal distribution is proposed to be a neural correlate of familiarity-based recognition. The late old/new effect (post-500 ms) is supposed to reflect memory recollection processes.

**Results:**

OCD patients were reported to show a normal early old/new effect and a reduced late old/new effect compared to normal controls. In our study, OCD patients with a high virus load (group H) displayed exactly these effects, while patients with a low virus load (group L) did not differ from healthy controls.

**Conclusion:**

These results confirmed that OCD patients had impaired memory recollection processes compared to the normal controls which may to some extent be related to their BoDV-1 infection.

**Supplementary Information:**

The online version contains supplementary material available at 10.1186/s12888-022-04208-3.

## Introduction

Obsessive–compulsive disorder (OCD) is characterized by recurrent obsessions and compulsions which are time consuming, significantly interfering with daily living of the patient [1]. A disturbance of cortico-striato-thalamo-cortical (CSTC) regulatory loops has been repeatedly demonstrated by brain-imaging studies [2–5]. Additionally, the characteristic symptoms of OCD including chronic doubts, repetitive checking, ruminations, decreased behavioral flexibility and ritual behavior [6] have been related to neuropsychological deficits in the areas of learning and memory [7–9], visuospatial processing [7, 10] and executive functions [11–13].

A strategic memory deficit has been shown in verbal memory tasks [14–17]; however, more recent reports showed deficits on non-verbal memory [8, 18–20]. It has been pointed out that memory decisions in OCD patients are usually given with reduced confidence [21–25]. This phenomenon is known as *memory bias* [21, 22], thought to be responsible for the peculiar insecurity in OCD patients.

Event-related brain potentials (ERPs) studies reported the existence of abnormal sensory and cognitive information processing in patients with OCD [23, 26–30]. ERPs are tiny voltage fluctuations, which can be recorded non-invasively from the intact human scalp and have been widely used in studying memory [31–33] due to its high temporal resolution. Until now, there were few ERP reports of word memory recognition (the old/new effect) in OCD [34–37]. These studies revealed that the old/new effect has distinct topographical areas – the ‘parietal’ and ‘mid-frontal’ in relation to memory, namely recollection and familiarity [38]. Our previous ERP study demonstrated that severely ill OCD patients showed an impaired late old/new effect (conscious recollection) of the word memory compared to the control group, which suggested an impairment of working memory capacity in these patients due to the dysfunction in the frontal and cingulated cortex [36].

The aim of this study was to investigate a possible modulation of word recognition memory in OCD patients with Borna disease virus 1 (BoDV-1) infection. BoDV-1, an unique RNA virus with a non-segmented single strand genome of negative polarity [39, 40], dominates the species *Mammalian 1 bornavirus* within the family *Bornaviridae* [41]. BoDV-1 strains are characterized by a broad host spectrum [42], high genomic conservation (> 95% homology) of either animal or human strains [43] and global occurrence [44]. Human isolates could be retrieved from peripheral mononuclear cells (PBMCs) [45] and brain [46] of psychiatric patients. One isolate was originating from a patient with chronic OCD [45] and recently completely sequenced [43]. BoDV-1 infection has been further discussed in relation to OCD [29, 47, 48]. Recently, rare cases of human encephalitis attracted attention [49–53], resembling lethal forms of encephalomyelitis in animals [42, 54, 55]. However, clinical outcomes (encephalitis vs. psychiatric disease), and epidemiology (zoonotic vs. human-to-human transmission) are still controversial [44, 56]. Likewise, which diagnostic method should be applied remained a matter of debate.

A variety of different methods to detect BoDV-1 infection had been developed in the past 30 years. Among them, BoDV-1 specific antibodies in serum via immune fluorescence (IFT) played a key role [57], but were complemented by direct viral components (proteins, RNA) in PBMCs and to a small extent in cerebrospinal fluid of psychiatric patients [47, 48, 58–63]. Isolation of infectious virus from RNA-positive PBMCs [45] and significantly higher frequencies of antibodies and/or RNA in psychiatric patients vs. healthy individuals have suggested a contributory role for Borna virus in the etiopathology of psychiatric disorders (reviewed by [47, 63]). Interestingly, BD virus recovered from a psychiatric patient was susceptible to amantadine [64]. Human isolates from a patient with bipolar depression and an OCD patient both were functionally different from laboratory viruses in vitro [43, 65]. Notably, in a double-blind placebo-controlled trial, BoDV-1 infected patients with major depressive and bipolar disorder largely benefitted from effective antiviral treatment with amantadine [66], thereby confirming previous open trials [67, 68]. Amantadine both provided anti-depressive and antiviral efficacy, as viral antigens and antibodies declined in parallel to clinical improvements, thereby strengthening the view on a viral involvement of the virus in neuropathology resulting in psychological events [66]. It was hypothesized from animal models that high amounts of free antigens are prerequisites for an interference of viral components with neurotransmitter systems, most probably with kainite KA1 receptor of glutamate [55, 69]. The impact of BoDV-1 infection on the sensitive balance of neuronal circuits and altered bioelectric activities in neurons, thus disturbing cognitive processes and memory, have been frequently demonstrated by molecular means and animal experiments [59, 70–75]. Persistence of high amounts of CICs which are circulating antigen–antibody complexes, and plasma antigen (pAG) correlated with the severity of depression [48, 66, 76]. Furthermore, higher CIC levels were associated with attention-related cognitive changing (increased ERP N1 component) in OCD patients, in particular in secondary visual areas and the anterior cingulated gyrus [29].

Notably, experimental evidence also demonstrated that BDV P-protein alone could disturb developmental processes in the brain [77] and impaired memory formation [74, 78]. Memory impairment has also been reported in other viral infection such as Theiler’s murine encephalomyelitis virus [79], Langat virus [80] and Herpes simplex virus [81]. Based on the likely contribution of BoDV-1 infection to memory dysfunction, this study aimed to investigate whether memory problems were modulated in BoDV-1 infected OCD patients using ERPs.

## Methods

### Subjects

The local ethical committee at the Hannover Medical School, Hanover, Germany, approved the study (see Additional file 1: study history and disclaimer). Written informed consent was obtained from all participants. Twenty native German speakers fulfilling the DSM-IV criteria [1] for OCD, participated in this study. The patients' pre-study treatment strategies were not changed during the study. Patients with comorbid current psychiatric diagnosis, drug abuse, medical or neurological disorder including tics or Tourette’s syndrome were excluded from the study. Four OCD subjects had to be excluded because of blink or movement artifact. Characteristics of the subjects (*n* = 16) including demographic data and scores on the Yale-Brown Obsessive–Compulsive Scale (Y-BOCS) [82], the Hamilton rating scale for depression (HAMD, 21-items) [83], and the Beck Depression Inventory (BDI) were shown in Table 1.

**Table 1:** Characteristics of the obsessive–compulsive disorder group

Pat No	Age (y.)	Gen	Educ (y.)	Additional Medication (mg/d)	Y-BOCS (1–5/6–10)	HAM-D	BDI
1	35	F	13	Fluoxetine 60	18(5/13)	8	28
2	35	F	14	Fluoxetine 60 Carbamazepine 300	26(9/17)	25	30
3	33	F	13	Fluoxetine 20	19(12/7)	6	7
4	35	F	13	None	15(0/15)	14	13
5	35	M	14	Fluvoxamine 150	26(15/11)	16	19
6	47	M	13	None	22(12/10)	14	14
7	31	F	13	None	23(13/10)	12	4
8	24	M	15	Paroxetine 20	24(15/9)	15	6
9	56	M	19	None	32(17/15)	14	4
10	29	F	13	Fluoxetine 60	16(0/16)	15	27
11	21	M	13	Citalopram 20	16(11/5)	32	33
12	32	M	12	Venlafaxin 300	27(16/11)	5	23
13	34	M	14	None	16(9/7)	7	4
14	31	M	16	Sertraline 50	23(9/14)	21	22
15	35	F	16	None	35(17/18)	29	31
16	20	F	13	None	20(7/13)	5	7
Mean ± SD	33.3 ± 8.8		14 ± 1.75		22.4 ± 5.86(10.4 ± 5.4/11.9 ± 3.8)	14.9 ± 8.3	17 ± 10.8

*Abbreviations: Gen* Gender, *Edu.(y)* Education year, *Y-BOCS* Yale-Brown obsessive–compulsive scale, *HAMD* Hamilton rating scale for depression, *BDI* Beck depression inventory

### BoDV-1 infection monitoring

BoDV-1﻿-infection was investigated by collecting anticoagulated blood. BoDV-1-specific circulating immune complexes (CICs) were detected by an enzyme immune assay (EIA) with specifically immobilized anti-p40/p24 monoclonal antibodies. Antibodies (Ab) and plasma antigens (pAG) were also measured using EIA, according to the published protocol [48] at the Robert Koch-Institute, Berlin, Germany during 2002–2005 (see Additional file 1: Study history and disclaimer). The values of CICs, pAG, and Ab were given in Table 2 as extinction values at 405 nm, measured in the starting dilutions for each test as indicated. Follow-up samples of study patients were tested at weeks 2, 4, 6, 12, and 16 to allow for evaluating the course of infection and the CIC values were given in Table 3. Based on previous longitudinal studies, the expressions of BoDV-1-specific CICs in the blood plasma are indicating an acute activated infection state.

**Table 2:** Virological results of the obsessive–compulsive disorder group

**H-group**	**Pat. No**	**CICs-Ext. (1:20)**	**pAG-Ext** **(1:2)**	**Ab-Ext** **(1:100)**	**Comment**
	1	187	56	44	
	2	280	70	157	
	3	445	9	305	
	4	179	25	157	
	5	230	11	57	
	6	1068	542	378	
	7	678	104	135	
	8	399	47	69	
	Mean ± SD	433.25 ± 305.70	108 ± 178.22	185 ± 117	
**L-group**	**Pat. No**	**CICs-Ext**	**pAG-Ext**	**Ab-Ext**	**Comment**^**a**^
	9	66	34	79	negative
	10	52	72	57	negative
	11	154	7	84	
	12	58	4	71	negative
	13	137	45	181	
	14	96	48	198	
	15	164	51	60	
	16	162	45	54	
	Mean ± SD	111.13 ± 48.51	38.25 ± 22.85	90.5 ± 63.99	
H- + L-group	Mean ± SD	272.2 ± 269	77.5 ± 131.1	137.7 ± 103.3	

*Abbreviations*: *Ext* Extinction values at 405 nm, *CICs* BoDV-1-specific circulating immune complexes in blood plasma, specific for BoDV-1 antigens p40/p24, *pAG* antigens (p40/p24) in citrated blood plasma, *Ab* Antibodies. The values of CICs, pAG and Ab are given as extinction at 405 nm × 1000, measured by enzyme immune assays (EIAs) in the starting dilution in brackets for each test (see Methods)

The cut off value of each test is ≥ 100. Results < 100 are regarded negative

H-group = patients with high CICs; L-group = patients with low CICs

^a^Comment: Patients 9, 10 and 12 were negative at the time of initial sampling (week 0) shown here but developed CICs on week 2, lasting to week 6 up to week 16 (see follow-up data, Table 3)

**Table 3:** Infection follow-up of the obsessive–compulsive disorder group based on circulating immune complexes (BoDV-1 CIC)

**H-group**	**Pat. No**	Week 0*	**Week 2**	**Week 4**	**Week 6**	**Week 12**	**Week 16**	**Level**^**a**^
	1	187	80	54	70	246		Discontinuous
	2	280	323	137	80	74	154	Discontinuous
	3	445	136	299		376	29	Continuous
	4	179	172	201	70	43		Continuous
	5	230	19	76	48	66	169	Discontinuous
	6	1068	815	856	513	1060	468	Continuous
	7	678	662	515	624	309		Continuous
	8	399	165	362	258	434	381	Continuous
**L-group**	**Pat. No**	Week 0*	**Week 2**	**Week 4**	**Week 6**	**Week 12**	**Week 16**	**Level**
	9	66	132	145	145	54	47	Discontinuous
	10	52	223	209	181	94	35	Discontinuous
	11	154	302		155	368	218	Continuous
	12	58	140	160	193	170	214	Continuous
	13	137	326	193	215	286		Continuous
	14	96	142	200	205	128		Continuous
	15	164	111	355	61	81	117	Discontinuous
	16	162	132	67	89	151		Discontinuous

*CICs* = *BoDV-1*-specific circulating immune complexes in blood plasma, specific for BoDV-1 antigens p40/p24. The values of CICs are given as extinction at 405 nm × 1000, measured by enzyme immune assays (EIAs) at the starting dilution of 1:20 for each sample (see Methods). *The initial sampling was at week 0 and represented the basic virological results for H- and L-group patients in this study (see Table 2)

The cut off value of each test is ≥ 100. Results < 100 are regarded negative. H-group = patients with high CICs; L-group = patients with low CICs. ^a^Level: “Discontinuous” means changing activity levels of infection within 16 weeks (last sampling). “Continuous” means stable activity levels of infection at least for 4 weeks, up to 16 weeks (last sampling)

Two subgroups of patients were formed with regards to BoDV-1-specific CIC levels given in Table 2 (initial sample, week 0): group H (for “high”; *n* = 8, 5 women) with relatively high levels of BoDV-1-specific CICs and group L (for “low”; *n* = 8, 3 women) with relatively low levels. Psychometric and virological parameters of the two subgroups were shown in Table 4. Twelve healthy control subjects (7 women, aged 20–48 years, mean age ± SD, 33.2 ± 10.3) were recruited from the student population and the staff of the Hannover Medical School and were matched closely with respect to age, education and handedness. They were diagnosed and found to be not infected with BoDV-1 (data not shown). There was no significant difference of age between OCD patients and the control group (T = 0.02; *p* = 0.99) and among OCD group H, group L and the control group [F(1, 2) = 0.10; *p* = 0.91].

**Table 4:** Characteristic comparisons between the two OCD groups with higher BoDV-1-specific CIC levels (group H) and with lower BoDV-1-specific CIC levels (group L)

Mean ± SD	**Group H** **(*n* = 8)**	**Group L** **(*n* = 8)**	**T value**	***p* value**
**Age**	34.4 ± 6.3	32.3 ± 11.1	0.22	0.65
**Edu. years**	13.5 ± 0.76	14.5 ± 2.32	1.16	0.27
**Y-BOCS (1–10)**	21.6 ± 4.0	23.1 ± 7.5	0.25	0.63
**Y-BOCS (1–5)**	10.1 ± 5.2	10.8 ± 5.9	0.05	0.83
**Y-BOCS (6–10)**	11.5 ± 3.3	12.4 ± 4.5	0.20	0.66
**HAMD**	13.8 ± 5.7	16.0 ± 10.5	0.28	0.60
**BDI**	15.1 ± 9.9	18.9 ± 12.1	0.46	0.51
**CICs-Ext.(1:20)**	433.3 ± 305.7	111.1 ± 48.5	8.7	***0.01***
**pAG-Ext.(1:2)**	108.0 ± 178.2	38.2 ± 20.6	0.92	0.16
**Ab-Ext.(1:100)**	185.0 ± 117.0	90.5 ± 64.0	4.02	0.07

(Abbreviations of the index see Tables 1,2 and 3)

### Stimuli and procedure

Three hundred German words (150 nouns, 86 verbs and 64 adjectives) were selected according to Meier [84] and the Beck inventory [85]. Mean log-frequency was –3.05. Word length varied between 4 and 11 letters (mean 6.7), subtending a visual angle of 1.7° vertically and 3.0° horizontally. Five randomized word lists of approximately equal length were constructed. Within each list, 83.3% of the words were repeated after 10 to 15 intervening items. Words were repeated only once. Each word was presented at the fixation point on a computer monitor located 110 cm from the subject.

The subjects´ task was to discriminate between first- and second- word presentation by pressing one of two buttons with the left or right index finger, while giving equal importance to speed and accuracy. The button assignment was counterbalanced between subjects. The words were presented serially in white color on a uniform dark blue background of a video monitor with duration of 300 ms (milliseconds) each with an inter-stimulus interval of 2 s (± 0.3 s). Subjects were requested and trained to fixate a dot in the center of the screen and not to move their eyes.

### Recording

The EEG was recorded from 31 electrodes including all standard sites of the International 10/20 system [84]. All electrodes were referenced to the right mastoid. The horizontal EOG was recorded bilaterally with electrodes at the outer ocular canthi and the vertical EOG was registered using an electrode below the right eye referenced to the right outer ocular canthus. The signals were amplified using 10 s time constant and processed with a band pass filter between 0.1 and 100 Hz (half amplitude low and high frequency cut-offs), digitized at a rate of 250 Hz and stored on the hard disk. Automated artefact rejection was performed off-line to eliminate data epochs contaminated by blinks, saccades and muscle activity and amplifier saturation. The mean rejection rate was 16% (7–28% for individual subjects).

### Data analysis

ERPs were averaged off-line (BrainVision Analyzer Version 2.0) for correctly detected first and second presentations of the words for each session and participant, as analyzed previously [27, 86]. The ERPs and behavioral performances were analyzed using repeated measure analysis of variance (ANOVA) using a 5% confidence level. Behavioral data were quantified by measuring reaction time (RT, ms) and hit rates (HR, %) (Pressing the correct button for first and second presentation).

ERPs were quantified as mean amplitudes in specified time windows. These data were entered into repeated measures’ analyses of variance (ANOVA). Separate ANOVAs were conducted for F3, Fz, F4 and C3, Cz, C4 in the time range of 250–500 ms (early old/new effect), for C3, Cz, C4 and P3, Pz, P4 in the time window of 450–650 ms, and for F3, Fz, F4 and C3, Cz, C4 from 650- 950 ms (both time windows for the late old/new effect), due to the repetition effect tending to be largest over frontal and parietal areas [87]. The ANOVAs comprised of the factors group (OCD patients vs. control group; patient’s group H vs. group L vs. control group), repetition (first vs. second presentation), and electrode location (left vs. middle vs. right). The factor group was treated as a between-subject variable, whereas the other factors were within-subject factors. All analyses were adjusted for non-sphericity with the Greenhouse–Geisser epsilon coefficient [88] whenever more than 1 degree of freedom was present in the numerator.

## Results

### Virological results

OCD patients were virologically categorized according to high infection values based on CICs (H-group, patients 1–8) and low infection values (L-group, patients 9–16). As shown in Table 4, the mean CIC values of the H-group were 433.25 ± 305.7 which differed significantly from those of the L-group (111.13 ± 48.51, *p* < 0.01). Within the L-group were three patients (No. 9, 10, 12) who turned out to be negative at the time of sampling (week 0), because of values below the cut -off of 100 in all three tests. However, these patients developed CICs on week 2, lasting until week 6 (No. 9 and 10) up to week 16 (No. 12) (Table 3), thus confirming infection. Table 3 further clarified that activation levels (CIC) could be stable for a long time (at least 4 weeks up to 16 weeks), as suggested in continuously symptomatic OCD. Continuous CIC levels accounted for more than half of our OCD patients, namely for 9 out of 16 patients (56.3%), but discontinuous virus activation was also found (7 out of 16 patients; 43.7%). Of the healthy volunteers, all tested negative for CICs, pAG and Ab.

### Behavioral results

Reaction times (RTs) to repeated words were significantly longer than to first presentations in the OCD patients and the control group [F (1, 26) = 53.35, *p* < 0.0001]. There was no interaction of group X repetition in either the comparison of the whole patient sample to controls [F (1, 26) = 1.41, *p* = 0.25] or for the patient subgroups and the control group [F (2, 25) = 1.18, *p* = 0.32] (Table 5). Repeated words were classified less accurately than first presentations [F (1, 26) = 40.47, *p* < 0.0001]. There were no interaction effects of group X repetition for the comparison of the entire patient group and the control group [F (1, 26) = 0.93, *p* = 0.34] as well as for the analysis including the patient subgroups and the control group [F (2, 25) = 0.74, *p* = 0.48] (Table 5).

**Table 5:** Reaction times (RT, ms) and hit rates (HR, %) (mean ± SD) for correctly recognized words in the different patient and control groups

	**OCD patients**		**Group H**		**Group L**		**Control Group**	
	**FP**	**SP**	**FP**	**SP**	**FP**	**SP**	**FP**	**SP**
**RT**(ms)	607 ± 43	671 ± 46	590 ± 38	645 ± 44	623 ± 43	697 ± 33	610 ± 50	656 ± 32
**HR**(%)	96.0 ± 4.2	76.8 ± 13.6	95.0 ± 5.5	73.2 ± 12.4	97.0 ± 2.4	80.4 ± 14.5	93.9 ± 8.8	79.7 ± 10.8

*Abbreviations*: *FP* First presentation, *SP* Second presentation

### ERP results

**Fig. 1﻿:**
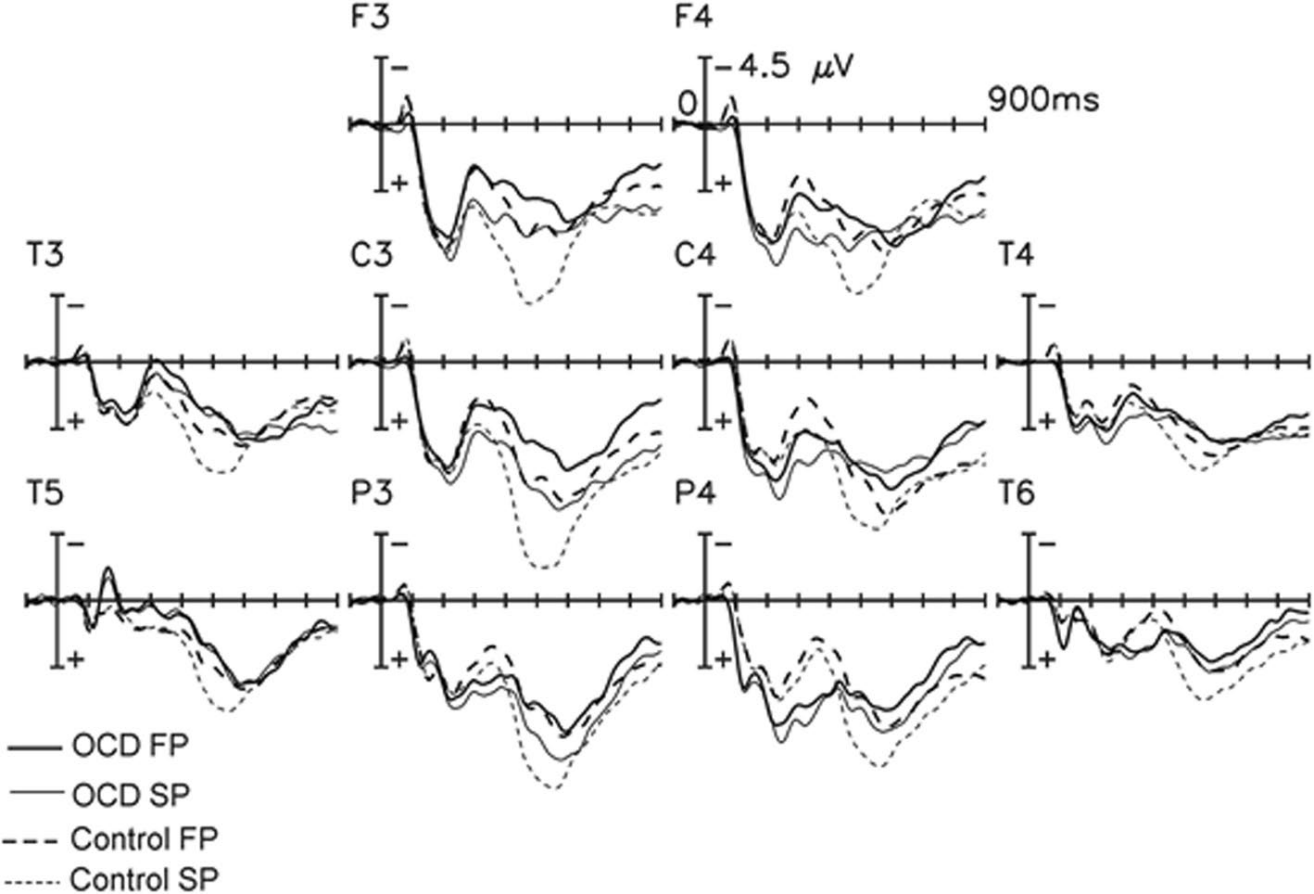
ERPs comparing OCD patients and controls. Grand average ERP waveforms to correctly detected first and second presentations of the words for the OCD group (*n* = 16) and the normal controls (*n* = 12) at F3, F4, C3, C4, P3, and P4. The second presentations of words elicited a more positive-shift starting at about 220 ms after the onset of the stimulus and extending for the rest of the recording epoch. This old/new effect is widespread and has a left frontocentral maximum. The patients showed reduced late old/new effect (ca. 450–650 ms) compared to the controls. FP = first presentation, SP = second presentation

The grand-average ERPs of the OCD patients (*n* = 16) and the control group (*n* = 12) elicited by correctly recognized first (new) and second (old) word presentations are shown for selected electrodes in Fig. 1. In both groups ERPs to repeated and correctly classified “old” items were more frequently positive than for new items, which represented the old/new effect. This old/new effect had an onset of about 220 ms post-stimulus. The early part of the old/new effect was indicated by the ERPs between 250 and 500 ms at the frontal electrodes for both groups [factor repetition: F (1, 26) = 34.78, *p* < 0.001]. But there was no difference for this early old/new effect between both groups [interaction group X repetition effect: F (1, 26) = 0.13, *p* = 0.73]. The ANOVA also revealed a significant late old/new effect with the centro-frontal maximum measured between 450 and 650 ms (factor repetition in both groups at the central electrodes: [F (1, 26) = 13.15, *p* < 0.01]. This effect appears to be more pronounced over the left side for both groups (an interaction of the factors, repetition X electrode [F (1, 26) = 10.27; *p* < 0.01]. There was no interaction group X repetition effect [F (1, 26) = 1.3, *p* = 0.25] and no main group differences [F (1, 26) = 2.18, *p* = 0.15] of the mean amplitudes between both groups at the central electrodes. But the single electrode statistical analysis showed the group difference at C3 [F (1, 26) = 4^**.**^27, *p* < 0.05], not at Cz [F (1, 26) = 1^**.**^55, *p* = 0.23] and at C4 [F (1, 26) = 1.08, *p* = 0.31]. From 650 to 950 ms, the factors of the within-group or between-group ANOVA showed no difference between both groups. The followed individual analysis for each group disclosed that the OCD group displayed no late old/new effect at the parietal electrodes, but at C3, as compared to the control group (Table 6).

**Table 6:** ANOVA results for the old/new effect (*Repetition*) and its interactions with the factor *electrode* (in parentheses) during two latency intervals at the different electrode sites for OCD group, group H, group L, and the control group

		**OCD**	**Group H**	**Group L**	**Controls**
250–500 ms	F3/Fz/F4	5.37(0.03)	13.08(0.009)	0.09(0.77)	18.99(0.001)
	C3/Cz/C4	6.97(0.02)	13.95(0.007)	0.27(0.62)	8.22(0.01)
450–650 ms	C3/Cz/C4	3.07(0.10)	7.56(0.03)	0.39(0.55)	15.23(0.002)
	P3/Pz/P4	2.30(0.15)	4.624(0.069)	0.47(0.51)	18.39(0.001)

**Fig. 2﻿:**
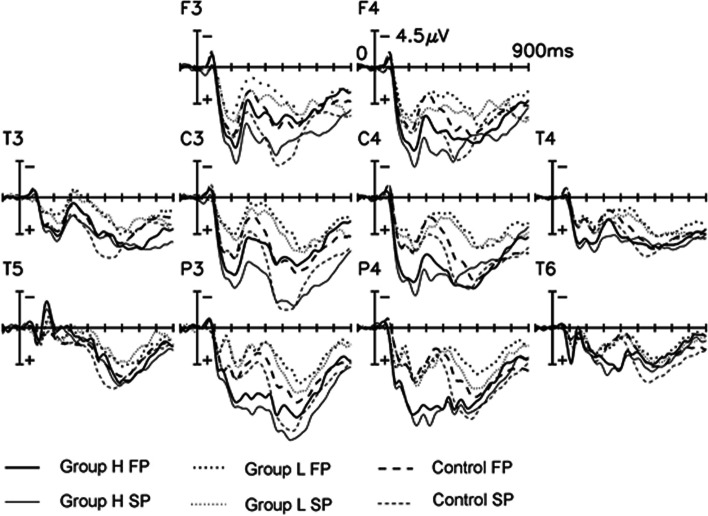
ERPs comparing OCD groups H and L and controls. Grand average ERP waveforms to correctly detected first and second presentations of the words for the OCD group H (*n* = 8), group L (*n* = 8) and the normal controls (*n* = 12) at F3, F4, C3, C4, P3, and P4. Group H showed early old/new effect (ca. 250–500 ms) and partially late old/new effect only over the left hemisphere in contrast to controls. Significant early and late old/new difference can be observed for the whole recording epoch between group L and the controls. FP = first presentation, SP = second presentation

Figure 2 disclosed the grand-average ERPs for the OCD group H (*n* = 8), OCD group L (*n* = 8) and the control group (*n* = 12) at the selected electrode sites. For the early portion of the old/new effect (250–500 ms post-stimulus), there was a significant repetition effect [F (1, 25) = 25.37, *p* < 0.0001], interaction effects of repetition X electrode [F (2, 25) = 8.08, *p* = 0.004], and repetition electrode X group [F (2, 25) = 3.3, *p* < 0.05]. In these three groups, the early old/new effect differed between groups for the different electrodes. The between-group differences of the mean amplitudes were present at the different electrodes. There was a significant difference observed for the between-group effect at the central areas. The pair-wise comparison of any two groups showed that the ERP amplitudes were significantly more frequently positive in the group H than in group L and control group at the central area. No significant differences were found between group L and the control group (Table 7). As shown by the individual analysis for each group, the control group and group H showed the early old/new effect at the fronto-central electrodes, while group L did not (Table 6).

**Table 7:** Between-subject differences of mean amplitude measures for the ERP components at the different electrode sites for group H, group L and the control group

	**Electrode**	**Group H Vs. Group L Vs. Control**		**Group H** **Vs. Group L**	**Group H Vs. Control**	**Group L Vs. Control**
		F(2,25) value	*p* value	*p* value	*p* value	*p* value
**250–500 ms**	F3/Fz/F4	3.221	0.057	***0.018***	0.21	0.148
	C3/Cz/C4	5.87	***0.008***	***0.002***	***0.033***	0.156
**450–650 ms**	C3/Cz/C4	8.301	***0.002***	***0.001***	0.627	***0.002***
	P3/Pz/P4	5.813	***0.008***	***0.003***	0.202	***0.026***
**650–950 ms**	F3/Fz/F4	0.638	0.537	0.416	0.283	0.850
	C3/Cz/C4	0.836	0.445	0.210	0.556	0.4424

From 450 to 650 ms, the within-group ANOVA disclosed the late portion of the old/new effect to be statistically significant [at the central area, F (1,25) = 10.99, *p* < 0.01]. The difference of the late old/new effect between groups was more pronounced over the left hemisphere. The central areas were indexed by an interaction of the factors, repetition X electrode X group [F (2, 25) = 4.77, *p* < 0.05]. Only at C3, the single electrode statistical analysis showed that there is an interaction effect of repetition X group [F (2, 25) = 3.24, *p* = 0.05]. These effects still remained at the central areas in the later interval of 650–950 ms [the interaction effect of repetition X electrode X group: F (2, 25) = 4.563, *p* = 0.01; the interaction of repetition X group at C3: [F (2, 25) = 3.669, *p* = 0.04]. As seen in the individual group analyses (Table 6), group H showed the late old/new effect only at the left centro-parietal areas in contrast to the control group (bilateral) while group L showed none. In addition, there were significant differences of the mean amplitudes between these groups at the centro-parietal electrodes (Table 7). The pair-wise comparison of any two groups disclosed that the ERP amplitudes in group L were much smaller than in group H and the control group at the centro-parietal areas. No significant differences were found between group H and the control group (Table 7).

## Discussion

The aim of the present study was to 1) investigate changes of the ERP old/new effects in the continuous word recognition in OCD patients in contrast to normal controls and, 2) explore the potential correlation of BoDV-1 infection and memory effects. The behavioral data showed a significant effect of the old word recognition on reaction times and target hit rates with less performance than for new words. But this effect elicited no differences between OCD patients and normal controls or between the OCD subgroups and the normal controls. This clarifies the fact that the groups did not differ in their tradeoff between response processing favoring one or the other parameter [27]. It supports the notion that group differences in ERP measures are related to the pathophysiological basis of OCD.

### ERP findings in OCD patients

The﻿ ERPs revealed two major findings. First, the early effect of word repetition resulted in an increased positive ERP waveform to repeated words in the time range between 250 and 500 ms post stimulus, with a maximum effect at the fronto-central areas. This effect showed no difference between the whole OCD group and the normal controls. Interestingly, group H (with high levels of BoDV-1-specific CICs) had similar early old/new effects with higher amplitudes compared to the control group. However, group L (with low levels of BoDV-1-specific CICs) had no early old/new effect at this time window. Rugg and colleagues [89, 90] stated that the early old/new effect differed according to whether items were old or new, but was insensitive to accuracy of recognition judgment and the depth of manipulation processing. Moreover, the lack of sensitivity reflected neural activity associated with a form or forms of memory (familiarity) distinct from recollection [90]. Our results suggested that the whole OCD group and group H reflected relatively normal familiarity-derived recognition processing in comparison to the controls while group L alone had a lack of this effect.

The second noteworthy aspect of the ERP findings concerned the memory effect that occurred from approximately 500 ms after the stimulus, which differed from the early old/new effect. The scalp topography was strongly left centro-parietal from 450 to 650 ms and this memory effect is sensitive to depth of processing (Remember or Know), which reflects neural activity associated with the conscious recollection of test items’ study episodes, as shown in other studies [38, 89–91]. In our study, the whole OCD group exhibited reduced late old/new effect in the latency of 450–650 ms in contrast to normal controls, but no difference was found in both groups from 650 to 950 ms. Rugg and Curran [38] suggested that ‘no difference’ may be due to small or non-existent in a ‘simple’ word recognition test. One can argue that the decreased old/new effect in the OCD group reflected an impaired conscious recollection of these words compared to healthy controls. What’s more, OCD subgroups showed sufficiently distinct ERP waveforms regarding to this late old/new effect. Only at the left centro-parietal areas this effect was found in group H while group L showed none. This suggested that the two OCD subgroups with different BoDV-1 infection activity levels reflected different memory recollection processing. However, one should be cautious about linking the current impaired memory processing in OCD with interpretations regarding neurocircuitry abnormalities and the infection levels of BoDV-1. This will be discussed at the end of this section.

Kim and colleagues [34] investigated the word memory in OCD using an ERP word recognition task study. Their results demonstrated that the controls showed the old/new effect during the 200–500 ms period post-stimulus, while the OCD patients did not. The absence of an old/new effect on ERP in OCD patients was discussed in terms of dysfunction of the fronto-striatal system, which plays an important role in OCD. Furthermore, Kim and colleagues [35] studied cortical source localization of word repetition effects (old/new effects) in patients with OCD by employing the equivalent current dipole model. Their results suggested that OCD patients suffered from the encoding deficits in the word processing, particularly in the left hemisphere.

The maximum recollection-sensitive effect of word repetition was seen obviously over the left electrodes in our study. This was in accord with the results of numerous other studies [89, 92, 93]. Wilding and Rugg [94] further noted that an effect in the asymmetric scalp distribution is due to a generator lateralized to the left hemisphere, and the left hemisphere is important for many aspects of verbal memory. Likewise, neuroimaging data showed that the left hippocampal formation along with regions of left temporal and frontal cortex involves in late old/new effect (recollection) [90]. These findings support the current ideas about the role of the hippocampal formation in episode memory retrieval and provide complementary information about the localization and time course of the cortical correlates of the recollection of recently experienced words.

OCD patients are at disadvantage, when they actively attempt to retrieve preciously encountered information, this is due to the impairment in non-verbal and verbal memory performance that is correlated with the deficits in semantic clustering and organizational strategies [14, 17]. Active organization of verbal or non-verbal information during encoding is thought to rely on a set of higher-level control processes, called ‘executive functions’, that modulate more basic cognitive, motor or memory functions and rely on the integrity of frontostriatal systems [90]. This suggests that failure to initiate strategies spontaneously is a salient characteristic of executive dysfunction in OCD. Behavioral key features of OCD such as checking or rereading might be due to deficits in memory and structuring information [14]. We have also presented electrophysiological evidence that OCD patient group H showed disturbed conscious word recollection and may utilized an alternative mechanism to recognize words, which seemed to be closely related to the infection levels of BoDV-1 (CICs).

### BoDV-1 infection in animal models

BoDV-1﻿ is an evolutionary old pathogen, which is perfectly adapted to highly specialized neurons in mammals, especially in the limbic system [55]. BoDV-1 causes behavioral disturbances in animals, such as depression-like apathy or excitation, spatial discrimination memory deficit, and learning deficiencies which are likely to be the consequence of viral proteins’ interference with neurotransmitter balances in the limbic system [72, 75, 95] or synaptic vesicle recycling [96]. BoDV-1 infected rats have high levels of viral nucleic acid in the prefrontal cortex, and elevated levels of mesocortical dopamine activity [73]. Kamitani and colleagues [77] have reported that glial expression of BoDV-1 phosphoprotein (P-protein) alone, in the absence of infection, could induce behavioral and neurological abnormalities in transgenic mice. The transgenic brains exhibit a significant reduction in brain-derived neurotrophic factor, serotonin receptor expression, as well as a marked decrease in synaptic density. BoDV-1 infected rats have shown hyperactivity and stereotyped behaviors similar to those following neurotoxic or electrolytic lesions in frontal cortex or its catecholamine afferents in rats.

Rat models of neonatal BoDV-1 infection resulting in virus persistence have shown learning and memory deficits [72, 75]. The hippocampus has a predominant role for memory and learning [97–99]. Initial viral dissemination was observed on post infection day 28 in the astrocytes of subiculum and later in the hippocampal neurons. At day 65 post infection, the spread went from the CA1 to the CA3 region and was abundant. The intensity of infection was more pronounced at day 120 post infection in the dendrites of CA3 neurons [100]. Intra-axonal spread was shown to be the major route for virus dissemination [101]. The persistent infection of BoDV-1 in animals favors hippocampus and limbic structures as preferential areas of replication [54, 100–102]. The pathophysiology is observed as cortical shrinkage and degeneration of granule neurons of dentate gyrus [72, 103]. The spatio-temporal occurrence of BoDV-1 glycoprotein was observed in the cerebral cortex, hippocampus, amygdala and thalamus [104]. However, the over-expression of virus nuclear and phosphoproteins (N- and P-protein) present in the hippocampus of rats with learning deficiencies [75] supported the hypothesis of an interference of those major proteins at glutamate receptor sites, namely the kainate KA1 receptor [69]. Notably, N- and P-protein are the major diagnostic markers indicating an activated infection [47, 48]. The monoclonal antibodies used in the EIAs for the detection of CICs and antigen (pAG) in plasma were shown to recognize conserved conformational epitopes on N-protein and phosphorylated (active) P-protein [105]. In BoDV-1 infected rats, the synaptic plasticity of dendate gyrus neurons has been altered [71]. Persistent infection in the brain did not affect survival and morphology, but the synaptic plasticity of the hippocampal neurons [70, 78, 106, 107]. Learning and memory consolidation are based on the strength of the synaptic plasticity [108]. The evidence from animal models suggests that the synaptic plasticity was shunned by a complex interference of BoDV-1 infection, most probably mediated by major proteins N and P, in hippocampal neurons resulting in the inhibition of learning and memory consolidation.

PKC and CaMKII have an important role in the memory formation [109] and in psychiatric illness [110]. It is also understood that the BoDV-1 P protein needs PKC and CaMKII for its phosphorylation [111, 112] and viral spread [113]. Abrogating the PKC binding site of BoDV-1-P protein resulted in restorage of normal synaptic plasticity [114]. These research reports clearly stated that BoDV-1 P-protein utilizes secondary messenger signaling for its replication and spread. Recently, Betourne and colleagues [78] have shown BoDV-1 P protein alone can impair memory, like Kamitani and colleagues [77] demonstrated earlier via a transgenic mice model. This provided strong evidence that BoDV-1 infection, at least via its pathogenic P-protein, may hinder the formation of memory and memory recollection.

### BoDV-1 infection in human psychiatric patients

In﻿ humans BoDV-1 infection has been finally accepted because of recent cases of associated encephalitis [49–53, 115]. However, a contributory role to psychiatric disorders which display behavior and cognitive disturbances, is still under discussion but has longtime proponents, due to correlative evidence in numerous studies [44–48, 57–64, 66–68, 76, 86, 116, 117]. In this study, we demonstrated significant differences in the early and late ERP old/new effects between OCD patient group H and group L. The group H showed impaired old/new effects with higher mean amplitudes, while group L had no word old/new effects in any time window compared to the normal controls. The clinical characteristics of the patient group H and group L, for example, ages, educational years, Y-BOCS scores, and HAMD scores did not differ significantly. This suggested that the levels of BoDV-1 infection, rather than the severity of clinical symptoms [36] and clinical subtypes, were involved in the abnormal patterns of word memory processes in both OCD subgroups. This was the first demonstration that BoDV-1 activity (amount of CICs) could be correlated with disease-relevant and memory-related cognitive changes (impaired old/new effects) in humans. Only those OCD patients with high CIC levels displayed mnestic dysfunction, those with low levels did not, suggesting that BoDV-1 unfolds disease-related effects depending upon the state of infection and not at any time. This hypothesis is in line with fluctuating activity levels corresponding with disease episodes in major depression [48, 66]. Notably, our pre-study screening suggested that a major proportion of OCD patients are at risk of activating Borna disease virus 1 infection.

The increased general amplitude in group H patients could fit well with our previous study [29], where we found that OCD patients with high CIC levels showed hyperactive attention-related information processing (an increased N1 ERP component and larger P3b amplitude) compared to those with low CIC levels and controls. Indeed, functional neuroimaging studies strongly suggested that OCD involves hyperactivity of CSTC networks [118, 119]. It is interesting to know that the subiculum, a subdivision of hippocampus formation which lies in between the entorhinal cortex and CA1 subfield of hippocampus plays an important role in memory recollection [120]. Viral spread in animal models was observed from subiculum to the hippocampus and then to the cortex [100]. The present findings are consistent with such hyperactivity and support the hypothesis this may be modulated by BoDV-1 infection, possibly through interfering of viral components with neurotransmitters (e.g., glutamate and aspartate) and impairment of synaptic plasticity, especially at hippocampus areas.

## Conclusion

In conclusion, ERP data from this study have added weighs to the evidence in support of impaired memory information processing in OCD. Additionally, the present study, being the first study to correlate BoDV-1 infection parameters with memory functions in humans, provides a new insight into the suggested relationship of BoDV-1 infection with cognitive dysfunctions in OCD patients. This will motivate further investigations along this line. Follow up studies in OCD patients with continuous vs. discontinuous virus activation could be useful to clarify whether present or absent virus activity correspond to mnestic dysfunction levels. Subsequent studies, however, need to replicate these preliminary findings in a larger sample size and to further investigate the possible mechanisms of neurotropic BoDV-1 infection and possible treatments.

## Supplementary Information

**Additional file 1: Supplemental file 1.** Study history and disclaimer.

## Data Availability

All data generated or analysed during this study are included in this published article and its supplementary information files.
