# Supplementary material for: Word recognition memory and serum levels of Borna disease virus specific circulating immune complexes in obsessive–compulsive disorder
Source: BMC Psychiatry. 2022 Sep 8;22:597. doi: 10.1186/s12888-022-04208-3 (PMC9454108; doi:10.1186/s12888-022-04208-3)
Supplement: Supplementary file 1 — Additional file 1: Supplemental file 1. Study history and disclaimer. [file 12888_2022_4208_MOESM1_ESM.docx]

**Supplemental file 1: Study history and disclaimer**

**Study history**

Two decades of pre-publication history are unusual. However, no similar study has been conducted in the meantime which may have impaired our study’s novelty.

The core part of the clinical investigation including preparatory parts and basic virology was conducted between 2002 and 2005. The discontinuity in post-study evaluation of clinical and virological study parts, and manuscript versions was on the one hand due to shifting of workplace and/or area of activity of some study authors. On the other hand, publication was significantly delayed through doubts raised in the scientific community about human infection until final incontrovertible proof of human infection by BoDV-1 caused encephalitis cases published in 2018.

In the interdisciplinary study, psychiatrists (YZ, AAPA, HME, DED) of the Clinic of Psychiatry, Social Psychiatry and Psychotherapy, Hannover Medical School, collaborated with virologists of the Institute of Virology at the Free University of Berlin (FU Berlin) (HL) and of the Project Bornavirus Infections (LB) at the Robert Koch Institute (RKI), Berlin, Germany. DED has later moved to the Ameos Clinic Hildesheim Psychotherapy and Psychiatry, Germany (present address). HL has meanwhile the status of a professor emeritus at his home university. HME passed away on 16^th^ of September 2018 but approved a pre-final version of the manuscript. LB has left the RKI upon retirement (2015). She assigned to the Freelance Bornavirus Workgroup of Joint Senior Scientists which HL has founded after becoming professor emeritus.

**Disclaimer (LB)**

The article reflects the author’s opinion but not the opinion of the Robert Koch Institute (RKI). All virological work assigned to the study has been performed during run time of the project Bornavirus infections at RKI laboratories. The project was terminated upon RKI decision by 31st December 2005. Therefore, since 2006, any publications reflecting this former work included the above disclaimer and/or were affiliated to institutions independent of her tenure at RKI.

Upon retirement she has reassigned to an unaffiliated authorship (see present address).
